# Effectiveness of Chemical Compounds Used against African Swine Fever Virus in Commercial Available Disinfectants

**DOI:** 10.3390/pathogens9110878

**Published:** 2020-10-24

**Authors:** Małgorzata Juszkiewicz, Marek Walczak, Natalia Mazur-Panasiuk, Grzegorz Woźniakowski

**Affiliations:** Department of Swine Diseases, National Veterinary Research Institute, Partyzantów 57 Avenue, 24-100 Puławy, Poland; marek.walczak@piwet.pulawy.pl (M.W.); natalia.mazur@piwet.pulawy.pl (N.M.-P.); grzegorz.wozniakowski@piwet.pulawy.pl (G.W.)

**Keywords:** African swine fever, disinfection, ASF, biosecurity, virucidal effects, suspension test, in vitro infectivity

## Abstract

African swine fever (ASF) causes huge economic losses and is one of most dangerous diseases of pigs. The disease is known for almost 100 years, an effective vaccine or treatment is still unavailable, only proper biosecurity measures, including disinfection, are being applied, in order to prevent disease outbreaks. Eight active substances, i.e., formaldehyde, sodium hypochlorite, caustic soda, glutaraldehyde, phenol, benzalkonium chloride, potassium peroxymonosulfate and acetic acid, were tested, in order to confirm their effectiveness against African swine fever virus (ASFV). This specific selection was done based on the World Organisation for Animal Health (OIE)’s recommendation and previous disinfectant studies on surfaces. The result of our study shows that most of them inactivate the virus, in recommended concentrations. In order to reduce the cytotoxicity of the four substances, Microspin S-400 HR columns were applied, therefore making it possible to demonstrate four logarithms virus titer reduction. Sodium hypochlorite, glutaraldehyde, caustic soda and potassium peroxymonosulfate showed the best ASFV inactivation rates, achieving titer reductions over 5 logs. Despite microfiltration, the virucidal activity of formaldehyde was not assessable, due to its high cytotoxicity. Our results showed that cleaning is particularly important, because removal of the soiling provides improved effectiveness of the tested chemical compounds.

## 1. Introduction

Today, in the face of the Covid-19 pandemic, caused by severe acute respiratory syndrome coronavirus 2 (SARS CoV-2), nearly everyone knows what biosecurity and disinfection rely on. Moreover, despite intensive research efforts, a commercially available vaccine has not been developed against Covid-19 or African swine fever (ASF) [1]. The first one affects people, and the second affects animals, but both are very dangerous and can lead to fatal outcomes.

ASF, caused by the African swine fever virus (ASFV), is one of the most dangerous (up to 100% mortality) infectious viral diseases of pigs, mainly due to enormous socioeconomic consequences. The disease affects animals of the *Suidae* family, mainly domestic pigs, wild boars and warthogs [2,3]. The current disease epizootic has begun in 2007 in Georgia and since then, it has been spreading widely in Eastern Europe. In 2018 ASF emerged also in Southeastern Asia, being the world’s largest pork producer. From August 2018 to May 2020, the Chinese Ministry of Agriculture and Rural Affairs (MARA) confirmed 170 ASF outbreaks, which resulted in a 1.2 million pig loss, decimating Chinese pork production by more than 40% [4]. Poland, as one of the leading pork producers in Europe, has been struggling with ASF since 2014, and to date, in total, over 9000 cases and 361 ASF outbreaks have been registered (data on 11.10.2020) [5]. Up to the end of 2019, 48 countries have reported ASF in domestic pigs, with over 10,000 separate outbreaks, causing the death or euthanasia of more than 2.5 million animals [6]. With the persistence of ASF in China, experts anticipate a further herd reduction and an associated drop in pig meat production of 15–25%, in 2020 [7]. Due to its infectivity, lack of vaccinations, and responsibility for serious economic and production losses, disease is listed as a notifiable by the World Organisation for Animal Health (OIE).

ASFV is a member of the family *Asfarviridae* and the genus *Asfivirus.* It is a large, enveloped DNA-virus. Its virions have a specific icosahedral structure of a protein capsid, which surrounds an internal membrane and a nucleoprotein core [2,8]. The continuous spread of ASF is driven by direct contact with infected pigs (i.e., aerosol, fluids and excretions), by the bites of soft ticks belonging to the *Ornithodoros* genus and indirect contact through contaminated feed, pork meat, people, vehicles or fomites [9,10]. In the recent disease epizootic in Eurasia, wild boar plays a key role in pathogen transmission, as it is main reservoir in the environment [11]. Irresponsible human activity may also be involved in the transmission of the virus, especially when it comes to spread of the disease over long distances. Despite quite little is known about possible virus carriers, the risk of potential outbreaks mainly come from poorly implemented biosecurity measures, including amongst others: unchanged footwear and outerwear, swill feeding or buying pigs from unknown sources [12,13,14]. As already proven, ASFV is a very resistant to a wide variety of conditions. The virus’s tolerance to pH changes ranges from <3.9 to >13; in blood stored in refrigerated conditions, it will persist up to 18 months; in frozen meat, up to 1000 days; and in dried bacon, it is only 300 days. It proves the importance of the drying process on the virus survival ability [15]. 

Biosecurity is defined as the implementation of a set of measures that reduces the risk of infection, amongst other, through cleaning and disinfection [16]. The full process of cleaning and disinfection involves five essential steps: dry cleaning, wet cleaning, drying, disinfection and final drying. Due to the lack of a commercially available, safe and effective vaccine against ASF, it is very important to prevent disease introduction and spreading. Nevertheless, in the case of its occurrence, it is also important to bring affected facilities back into production, as safe and as soon as possible [16]. ASFV may remain infectious for a long time, in feces and blood, moreover in the presence of an organic matter, the virus might be even more stable and may survive longer [17]. Hence, choosing a suitable disinfectant and applying it effectively, taking into account environmental conditions, contact time, pH and temperature ranges, plays a crucial role in biosecurity. In order to improve the effectiveness of the measure, it is important to perform a cleaning step prior to proper disinfection, in order to get rid of organic matter [18].

The aim of the study was to prove the effectiveness of the following representative chemical compounds:

- Formaldehyde;
- Sodium hypochlorite;
- Caustic soda solution;
- Glutaraldehyde;
- Phenol;
- Chemical compounds based on lipid solvents, e.g., benzalkonium chloride;
- Multi-constituent compounds, e.g., potassium peroxymonosulfate;
- Organic acids, e.g., acetic acid, etc.

**Figure 1:**
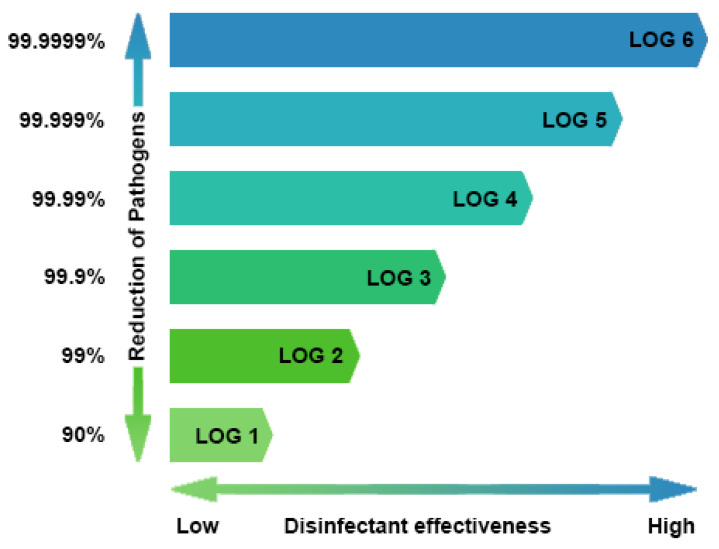
Logarithmic reduction in terms of percentage.

The above-listed compounds are widely recommended as being effective against ASFV and commonly used in the production of commercial disinfectants. Despite the extreme importance, little information is available regarding the in vitro suspension testing of disinfectants against ASFV [19]. Viruses may be classified into two groups: enveloped (more sensitive to most disinfectants) and non-enveloped viruses (much more resistant). As ASFV belongs to the enveloped group, OIE recommendation is based on the disinfection study results of other enveloped viruses, e.g., ORF virus (OV), equine viral arteritis virus (EVAV), pseudorabies virus (PRV), porcine reproductive and respiratory syndrome virus (PRRSV), and classical swine fever (CSF) [20,21,22]. In addition, several studies regarding the activity of selected active substances against ASFV, on various types of surfaces, have been published [20,23,24,25,26,27]. The mentioned articles concerned tests on surfaces where the virus was subjected to a drying process, which process always reduced the control virus titer. This repeatedly made impossible to get necessary 4 log difference, which is required to qualify the disinfectant as effective. The question is whether the reduction in viral titer is due to the disinfectant or perhaps the effect of drying process. In addition, the test method on surfaces has not been accepted yet as a procedure qualifying disinfectants as effective in the veterinary sector. Each virus should be considered as unique, in terms of its resistance to physical and chemical treatment. ASFV sensitivity to disinfection is worth individual testing inspired by the PN-EN 14675:2015 European Standard, where the ECBO virus was replaced by ASFV and several minor modifications were done, to provide an appropriate cell culture and virus propagating conditions. The active compound was considered as effective, when it showed at least a 4-log_10_ reduction in the initial viral titer, which is equivalent to a loss infectivity level of 99.99% [28] (Figure 1). 

## 2. Materials and Methods 

### 2.1. Cells and Viruses

The Vero-adapted BA71V strain was obtained from the European Union Reference Laboratory, in Spain. A Vero cell line was obtained from ATCC (ATCC^®^ CCL-81^TM^) and was subcultured in a Minimum Essential Medium (GIBCO, Life Technologies, Carlsbad, CA, USA), a 10% Fetal Bovine Serum (FBS) (Gibco, Billings, MT, USA) and a 1% Antibiotic Antimycotic Solution (100×) (Sigma-Aldrich, St. Louis, MI, USA). The cultures were grown at 37 °C, in a humidified atmosphere of air containing 5% CO_2_. 

### 2.2. Virus Stock Preparation

Subconfluent monolayers of Vero cells were infected with 10^6^ tissue culture infectious dose (TCID_50_/mL) of the virus and were incubated at 37 °C, until a 100% cytopathic effect was observed, usually after 4–5 days. In order to provide a sufficient virus titer (at least 10^6.5^ TCID_50_/mL), allowing for demonstration of a 4 log reduction of titer, after disinfectant treatment, the obtained viruses were subjected to 3 freeze/thaw cycles and precipitated, using the following buffer: 20% Polyethylene glycol (PEG) and 2.5 M sodium chloride in a 2:3 buffer:virus ratio. The virus–buffer solution was agitated overnight at 4 °C, subsequently ASFV was pelleted by centrifugation at 13,000 *g* for 90 min, at 4 °C, and resuspended in 1/10 volume of the initial medium. The obtained virus stocks were titrated and aliquoted, and stored at −80 °C. Virus titers were determined by a TCID_50_/mL titration, using the Spearman–Kärber method [29].

### 2.3. Disinfectants

Three selected concentrations of each active substance were prepared immediately before use by dilution in hard water. The basic concentrations were selected, based on OIE recommendation as being effective against ASFV [30]. Moreover, we have also tested one more concentrated and one more diluted solution of each agent.

We used the following concentrations: formaldehyde (POCH, Gliwice, Poland, CAS: 50-00-0): 0.4%, 0.8% and 1.6%; sodium hypochlorite (solution in water containing 15% active chlorine, Stanlab, Lublin, Poland, CAS: 7681-52-9): 0.3%, 1% and 1.5%; caustic soda (POCH, Gliwice, Poland, CAS: 1310-73-2): 1%, 2% and 3%; glutaraldehyde (25%, Carl Roth, Karlsruhe, Germany, CAS: 111-30-8): 0.1%, 0.5% and 1%; phenol (Chempur, Piekary Śląskie, Poland, CAS: 108-95-2) in 0.5%, 1% and 2%; benzalkonium chloride (Pol-Aura, Olsztyn, Poland, CAS: 63449-41-2): 0.5%, 1% and 2%; potassium peroxymonosulfate (Envolab, Długomiłowice, Poland, CAS: 70693-62-8): 0.5%, 1% and 2%; acetic acid (Chempur, Piekary Śląskie, Poland, CAS: 64-19-7): 1%, 2% and 3% concentrations.

### 2.4. Diluent and Interfering Substances

All tested chemical compounds were diluted with water, of standardized hardness, containing defined concentration of Mg^+^, Ca^2+^, Cl^−^, HCO^3−^ anions (pH 7). The hard water was prepared according to the PN-EN 14675:2015 European Standard. The suspension test was prepared with interfering substances: BSA—bovine albumin 3.0 g/L (low-level soiling) and BSA + YE—bovine albumin 10 g/L, plus yeast extract 10 g/L (high-level soiling) were prepared, according to the PN-EN 14675: 2015 European Standard.

### 2.5. Test Conditions

Each concentration was tested in triplicate. One part of the virus suspension was mixed with one part of the interfering substances, respectively, with low level soiling or high level soiling and incubated at 10 ± 1 °C for 2 min ± 10 s. Subsequently, eight parts of the disinfectant diluted to 1.25-fold of each tested concentration was added. The obtained mixture of virus, disinfectant and interfering substances was incubated at 10 ± 1 °C for 30 min ± 10 s; afterwards, the test tubes were placed on crushed ice (4 °C). The samples were immediately, serially diluted (in quadruplicates) 10-fold (both the control virus and experimental virus suspensions) in a Vero cell culture, in 96-well plates. The plates were incubated for 7 days, at 37 ± 2 °C, in air containing 5% CO_2_ and examined daily for the appearance of cytopathic effect (CPE). All plates were ultimately scored for cytopathic effect upon microscopic examination, for 7 days. A minimum 6.5 log_10_ (TCID_50_/_mL_) of virus titer in the control sample was required to demonstrate a ≥4 log reduction.

### 2.6. Cytotoxicity Reduction

Several chemical agents turned out to be cytotoxic to the Vero cells, therefore precluding in proper test performance and demonstration of a 4 log_10_ titer reduction. Microspin S-400 HR columns (GE Healthcare, Fairfield, CT, USA) were used, in order to remove the cytotoxic agent from the samples, right after 30 min incubation of the tested and control samples. Virus controls with and without micro-filtration were compared, to detect losses in the virus titer. 

### 2.7. Test Controls

Both standard and cytotoxicity controls were processed in the same manner as the chemical compounds, but the chemical agent was replaced with hard water. 

### 2.8. Statistical Analysis

Statistical analyses were performed, using the GraphPad Prism (version 8.4.3, La Jolla, CA, USA). Analyses of the mean differences between each disinfectant were shown with a standard deviation.

## 3. Results

The efficacy of chemical compounds was determined by comparing mean log reduction in the mixture of virus and tested substances, with the log titer of virus control. Disinfectant was considered virucidal, when a ≥4 log_10_ titer reduction, in comparison to the control, was demonstrated. A summary of the results of the first suspensions in vitro tests of chemical compounds are presented in Table 1.

Initial testing showed the high cytotoxicity of formaldehyde, glutaraldehyde, benzalkonium chloride and acetic acid, therefore the test was repeated, using Microspin S-400 HR filtration, and these results are included in Table 1. Microfiltration resulted in ≤0.2 log_10_ loss of the initial virus titer (data not shown); however, cytotoxicity was reduced from 1 log_10_ to ≥3 log_10_, therefore allowing for the assessment of the antiseptic effectiveness of the three compounds. Microfiltration was insufficient to reduce the cytotoxicity of formaldehyde, which showed the highest cytotoxicity in the initial testing.

**Figure 2:**
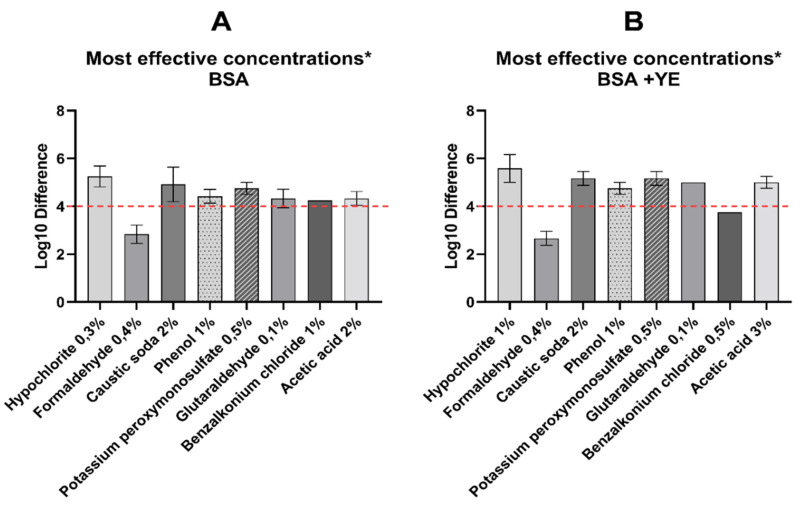
Most effective concentrations of tested disinfectants, in the presence of (**A**) low soiling level (BSA) and (**B**) high soiling level (BSA + YE). Maximum detectable log_10_ differences are presented. The red dashed line—virucidal effect threshold. * If the log difference was the same in one or more concentrations, the lower concentration was presented on the graph.

Initial testing of sodium hypochlorite (15% active chlorine) was performed at concentrations from 0.03%, 0.01% to 0.0075%, which correspond to 0.0045%, 0.0015% and 0.001125% of active chlorine, respectively, but none of these concentrations were effective (data not shown). On this account, the test of the sodium hypochlorite was repeated, using higher concentrations, e.g., 0.3%, 1% and 1.5% (0.045%, 0.15% and 0.225% active chlorine). This time, all concentrations, except 0.3% (at the high soiling condition) resulted in the virucidal effect, even reaching 5.58 (±0.47) mean log_10_ reduction (Figure 2). Sodium hypochlorite showed to be sensitive to the presence of organic material, therefore the higher concentration must be used, in order to provide the same disinfectant efficacy. Caustic soda, at the final concentrations of 1%, 2% and 3% caused ASFV inactivation, except the lowest concentration in the high soiling condition, which was ineffective. Despite filtration, 3% of the concentration in the low level soiling condition turned out to be cytotoxic and makes it impossible to evaluate effectiveness. Phenol, potassium peroxymonosulfate and benzalkonium chloride were examined in the same concentrations (0.5%, 1% and 2%). Three of these, were effective at 1% in both soiling conditions, except benzalkonium chloride, which worked virucidally, only at the low soiling condition. The highest phenol concentration induced a cytotoxic effect, while at 0.5% it was thoroughly ineffective. Glutaraldehyde showed its effectiveness under all conditions and concentrations, but unexpectedly, at 0.5% and low soiling, it was cytotoxic. Even in the case of unremovable cytotoxicity, most of the disinfectants reduced the ASFV titer by 3 logs_10_, which corresponds to 99.9% percent pathogen reduction (Figure 1). The greatest log reduction was observed in sodium hypochlorite.

**Figure 3:**
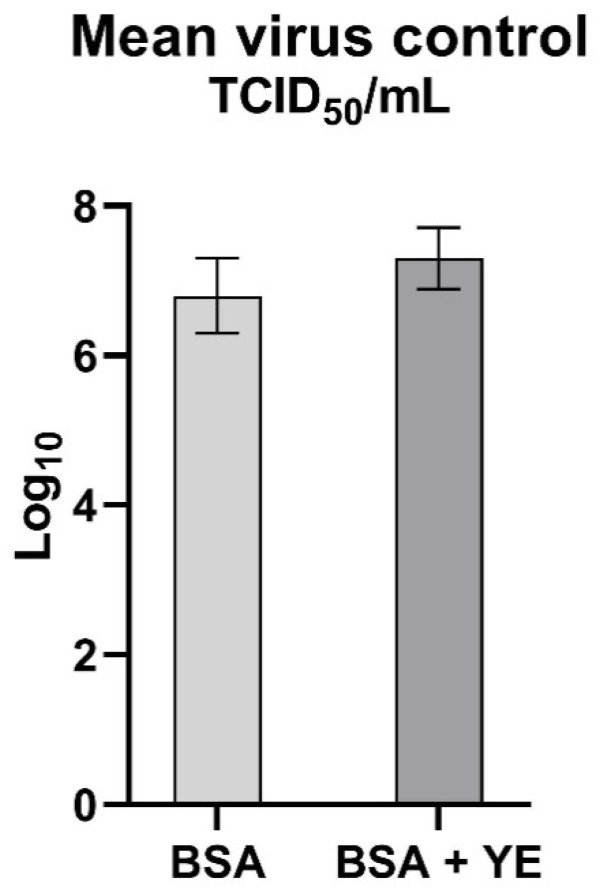
Mean titer of the virus control in two variants of soiling levels.

During our tests, it was observed that the control virus titer, under high-level soiling conditions, was significantly higher than that under low level soiling (Figure 3). It was, respectively, 7.3 (±0.41) and 6.8 (±0.5) TCID50/mL. This may prove that the virus replicates more efficiently in the presence of organic matter, which may affect assessment of the disinfectant. Thus, the key role of cleaning prior to proper disinfection should be emphasized.

## 4. Discussion

ASF is one of the most dangerous animal diseases, decimating pig populations almost worldwide; therefore, disinfection plays a key role in the prevention of the disease. Correct selection of a disinfectant and a properly applied disinfection process are crucial, to provide the highest effectiveness of the chemical substance, which depends also on the proper use, mechanism of action and application conditions, such as concentration, contact time, pH, temperature and the presence of organic matter.

To our knowledge, this is the first in vitro suspension test reporting virucidal activity of chemicals compounds against ASFV, based on a modified PN-EN 14675:2015 European Standard. 

Seven out of the eight tested chemicals (e.g., sodium hypochlorite, glutaraldehyde, caustic soda, potassium peroxymonosulfate, phenol, acetic acid and benzalkonium chloride) caused effective ASFV inactivation. Each of them was effective at specific concentrations, usually recommended by the OIE, and at different soiling conditions. Our studies showed that the control virus titer, in high soiling conditions, was higher in all tested compounds (Figure 3). It proves that the virus may replicate more efficiently in the presence of organic matter. Moreover, the organic matter may affect effectiveness of the disinfectant. It indicates that the key role of cleaning prior to proper disinfection should be emphasized. Higher virus titer in control under a high-soiling-level condition lead to a higher final difference, as can be seen in Figure 2. This confirmed Krug et al. studies, where the possibility of the influence of a higher serum concentration on survival and virus multiplication was noted [23], which showed slight discrepancies in the results obtained.

The virucidal efficacy of formaldehyde could not be determined, due to high cytotoxicity, which was not reduced by microfiltration. Nevertheless, ASFV titer reduction by 2.83 * (±0.31) at 0.4% concentration of formaldehyde was still observable, suggesting its moderate effectiveness. Nevertheless, using formaldehyde as a disinfectant has been limited, due to it is carcinogenicity, based on the International Agency for Research on Cancer (IARC) concluding that using it heightens the risks of nasopharyngeal cancer and leukemia. Glutaraldehyde showed very high virucidal efficiency, even at high soiling conditions, despite the cytotoxicity neutralization by microfiltration being required. Similar to previous reports, our study confirmed that glutaraldehyde, caustic soda and potassium peroxymonosulfate showed a wider ASFV inactivation spectrum [19,24,31]. Sodium hypochlorite is one of the most commonly used ingredients of disinfectants, because it is known to be effective even against hard-to-inactivate, non-enveloped viruses [32], bacteria and phages [33,34]. In our hands, this specific compound provided the biggest mean log_10_ titer reduction, i.e., by 5.58 (±0.47), thus being effective at almost every tested and presented concentration. The effectiveness of sodium hypochlorite has already been confirmed by Krug et al., during tests on surfaces, where, despite drying, 0.05% hypochlorite effectively inactivates ASFV [25]. Its effectiveness depends on the free active chlorine concentration in commercial, initial hypochlorite solution in tested samples and may depend on the bovine serum concentration used for cell sub-culturing [23]. Caustic soda and potassium peroxymonosulfate, which are widely used in commercially available disinfectants, showed the same highest mean reduction in viral titer, by 5.17 (±0.24) log_10_. Potassium peroxymonosulfate is the main active substance in commonly used disinfectants, approved by the OIE and the United States Department of Agriculture (USDA) [35,36]. Caustic soda is one of the strongest alkaline; therefore, the pH value of its tested concentrations reached >13 and was effective at almost every concentration of interest, probably due to the fact that ASF is susceptible to a pH less than 4 and over 13 [37]. With regard to acetic acid, it showed a pH > 3; therefore, it was fully effective at 3% and partially at 2% (only at low soiling conditions). Moreover, 1% phenol was virucidal in both soiling conditions, but the higher concentration was cytotoxic. Benzalkonium chloride, belonging to quaternary ammonium compounds (QUATs), inactivated ASFV only at 1% concentration, at low soiling conditions; moreover, it required prior filtration. 

Our previous studies of commercial disinfectants showed high cytotoxity in disinfectants based on QUATs and glutaraldehyde, which did not allow for proper result assessment [19]. Therefore, in this study, we confirmed the low virucidal activity of QUATs, which has recently been reported [20,38]. Other disinfectants showed similar results to the data obtained in the present studies. However, the cytotoxicity reduction practice introduced in the current research, allowed for confirmation of some chemical compounds efficiency against ASFV, which was not possible previously.

In summary, the highest virucidal activity against ASFV was shown in sodium hypochlorite, glutaraldehyde, caustic soda and potassium peroxymonosulfate. The wide range of effective disinfectants against ASFV is not surprising, since the virus belongs to the enveloped variant vulnerable to disinfectants interrupting its lipid-envelope structure. Moreover, the provided studies showed that ASFV may be also inactivated by pH manipulation, in the presence of a strong alkaline (caustic soda) or acid (acetic acid). Our research proved that the presence of organic matter is a crucial challenge, which may support the virus-replication process or lead to a reduction of the disinfection effectiveness. Therefore, cleaning with soap and water seems to be crucial, in order to reduce soiling and, therefore, improve disinfection efficacy cannot be omitted. In the presence of organic matter, ASFV multiplies more efficiently and is more resistant to inactivation. Despite using the proper disinfectants and appropriate contact time, the chemical compound may turn out to be ineffective.

In the future, it is worth getting involved in searching for eco-friendly disinfectants, showing reduced cellular cytotoxicity and a less harmful impact on humans and the environment.

## Figures and Tables

**Table 1:** Logarithmic reduction of African swine fever virus (ASFV) titer in the presence of tested active substances. Contact time, 30 min. Temperature of incubation, 10 °C. Each data point is the mean of three experiments, ±SD.

Active Substance	Tested Concentration	Log_10_ Difference ** (±SD) (TCID_50_/_mL_)		Virucidal Effect (Difference ≥ 4 Log_10_)	
		BSA	BSA + YE	BSA	BSA + YE
Sodium Hypochlorite	1.5%	5.3 (±0.35)	4.58 (±0.47)	Yes	Yes
	1%	5.3 (±0.35)	5.58 (±0.47)	Yes	Yes
	0.3%	5.3 (±0.35)	4.17 (±0.31)	Yes	No
Caustic Soda	3%	4.67 * (±0.77)	4.67 * (±0.51)	N/A	Yes
	2%	4.91 (±0.59)	5.17 (±0.24)	Yes	Yes
	1%	4.83 (±0.67)	4.17 (±0.31)	Yes	No
Phenol	2%	3.42 * (±0.24)	3.75 * (±0.20)	N/A	N/A
	1%	4.42 (±0.24)	4.75 (±0.20)	Yes	Yes
	0.5%	0.08 (±0.12)	0.08 (±0.12)	No	No
Potassium Peroxymonosulfate	2%	3.75 *(±0.20)	4.17 *(±0.24)	N/A	N/A
	1%	4.75 (±0.20)	5.17 (±0.24)	Yes	Yes
	0.5%	4.75 (±0.20)	5.17 (±0.24)	Yes	Yes
Acetic Acid ^c^	3%	4.33 (±0.23)	5 (±0.2)	Yes	Yes
	2%	4.33 (±0.23)	3 (±0.2)	Yes	No
	1%	3.92 (±0.12)	1.42 (±0.12)	N/A	No
Glutaraldehyde ^c^	1%	4.33 (±0.31)	4 * (±0)	Yes	Yes
	0.5%	4.33 (±0.31)	4 * (±0)	Yes	Yes
	0.1%	4.33 (±0.31)	5 (±0)	Yes	Yes
Formaldehyde ^c^	1.6%	2.08 * (±0.31)	1.67 * (±0.24)	N/A	N/A
	0.8%	1.83 * (±0.31)	2.5 * (±0.0)	N/A	N/A
	0.4%	2.83 *(±0.31)	2.67 * (±0.24)	N/A	N/A
Benzalkonium Chloride ^c^	2%	2.25 * (±0)	2.67 * (±0.12)	N/A	N/A
	1%	4.25 (±0)	3.75 * (±0)	Yes	N/A
	0,5%	4.08 (±0.11)	3.75 * (±0)	No	N/A

** Difference between control and tested sample. * Cytotoxic effect. N/A = the virucidal effect could not be evaluated, due to cytotoxicity. BSA = low soiling level (bovine serum albumin 3.0 g/L), BSA + YE; ^c^ cytotoxic effect was observable—results are presented after applying the cytotoxicity neutralization.

## References

[1] Gallardo C., Sánchez E.G., Pérez-Núñez D., Nogal M., De León P., Carrascosa A.L., Nieto R., Soler A., Arias M.L., Revilla Y. (2018). African swine fever virus (ASFV) protection mediated by NH/P68 and NH/P68 recombinant live-attenuated viruses. Vaccine.

[2] Arias M., De La Torre A., Dixon L.K., Gallardo C., Jori F., Laddomada A., Martins C., Parkhouse R.M., Revilla Y., Rodriguez F.A.J.-M. (2017). Approaches and Perspectives for Development of African Swine Fever Virus Vaccines. Vaccines.

[3] Mazur-Panasiuk N., Żmudzki J., Woźniakowski G. (2019). African swine fever virus—Persistence in different environmental conditions and the possibility of its indirect transmission. J. Vet. Res..

[4] Li X., Tian K. (2018). African swine fever in China. Vet. Rec..

[5] Pejsak Z., Niemczuk K., Frant M., Mazur M., Pomorska-Mól M., Ziętek-Barszcz A., Bocian Ł., Łyjak M., Borowska D., Woźniakowski G. (2018). Four years of African swine fever in Poland. New insights into epidemiology and prognosis of future disease spread. Pol. J. Vet. Sci..

[6] OIE Global Situation of ASF, 2019. African Swine Fever. https://www.oie.int/fileadmin/Home/eng/Animal_Health_in_the_World/docs/pdf/Disease_cards/ASF/Report_17._Global_situation_of_ASF.pdf.

[7] European Commission (2020). Short-Term Outlook for EU Agricultural Markets in 2020.

[8] Gallardo C., De La Torre A., Fernández J., Iglesias I., Muñoz M.J., Arias M. (2015). African swine fever: A global view of the current challenge. Porc. Heal. Manag..

[9] EFSA AHAW Panel (2014). Scientific Opinion on African swine fever. EFSA J..

[10] Mur L., Martínez-López B., Sánchez-Vizcaíno J.M. (2012). Risk of African swine fever introduction into the European Union through transport-associated routes: Returning trucks and waste from international ships and planes. BMC Vet. Res..

[11] Walczak M., Frant M., Juszkiewicz M., Mazur-Panasiuk N., Szymankiewicz K., Bruczyńska M., Woźniakowski G. (2020). Vertical transmission of anti-ASFV antibodies as one of potential causes of seropositive results among young wild boar population in Poland. Pol. J. Vet. Sci..

[12] Fasina F.O., Lazarus D.D., Spencer B., Brian T., Makinde A.A., Bastos A.D. (2011). Cost Implications of African Swine Fever in Smallholder Farrow-to-Finish Units: Economic Benefits of Disease Prevention Through Biosecurity. Transbound. Emerg. Dis..

[13] Woźniakowski G., Kozak E., Kowalczyk A., Łyjak M., Pomorska-Mól M., Niemczuk K., Pejsak Z. (2015). Current status of African swine fever virus in a population of wild boar in eastern Poland (2014–2015). Arch. Virol..

[14] Pejsak Z., Truszczyński M., Niemczuk K., Kozak E., Markowska-Daniel I. (2014). Epidemiology of African Swine Fever in Poland since the detection of the first case. Pol. J. Vet. Sci..

[15] EFSA AHAW Panel (2010). Scientific Opinion on African Swine Fever. EFSA J..

[16] FAD-PREP/NAHEMS (2016). NAHEMS Guidelines: Biosecurity.

[17] Stone S.S., Hess W.R. (1973). Effects of Some Disinfectants on African Swine Fever Virus. Appl. Microbiol..

[18] Sattar S. (2016). Cleaning, Disinfection, and Sterilisastion. IFIC Basic Concepts of Infection Control.

[19] Juszkiewicz M., Walczak M., Mazur-Panasiuk N., Woźniakowski G. (2019). Virucidal effect of chosen disinfectants against African swine fever virus (ASFV)—Preliminary studies. Pol. J. Vet. Sci..

[20] Shirai J., Kanno T., Tsuchiya Y., Mitsubayashi S., Seki R. (2000). Effects of Chlorine, Iodine, and Quaternary Ammonium Compound Disinfectants on Several Exotic Disease Viruses. J. Vet. Med Sci..

[21] Gallina L., Scagliarini A. (2010). Virucidal efficacy of common disinfectants against orf virus. Vet. Rec..

[22] Jeffrey D. (1995). Chemicals used as disinfectants: Active ingredients and enhancing additives. Rev. Sci. et Tech. de l’OIE.

[23] Krug P.W., Lee L.J., Eslami A.C., Larson C.R., Rodriguez L. (2011). Chemical disinfection of high-consequence transboundary animal disease viruses on nonporous surfaces. Biologicals.

[24] Krug P.W., Larson C.R., Eslami A.C., Rodriguez L.L. (2012). Disinfection of foot-and-mouth disease and African swine fever viruses with citric acid and sodium hypochlorite on birch wood carriers. Vet. Microbiol..

[25] Krug P.W., Davis T., O’Brien C., Larocco M., Rodriguez L.L. (2018). Disinfection of transboundary animal disease viruses on surfaces used in pork packing plants. Vet. Microbiol..

[26] Turner C., Williams S.M. (1999). Laboratory-scale inactivation of African swine fever virus and swine vesicular disease virus in pig slurry. J. Appl. Microbiol..

[27] Gabbert L.R., Neilan J.G., Rasmussen M. (2020). Recovery and chemical disinfection of foot-and-mouth disease and African swine fever viruses from porous concrete surfaces. J. Appl. Microbiol..

[28] Reybrouck G. (1998). The testing of disinfectants. Int. Biodeterior. Biodegrad..

[29] Hierholzer J., Killington R. (1996). Virus isolation and quantitation. Virol. Methods Man..

[30] OIE (2019). African Swiene Fever. Aetiology. World Organ. Anim. Health.

[31] Becker B., Henningsen L., Paulmann D., Bischoff B., Todt D., Steinmann E., Steinmann J., Brill F.H.H., Steinmann J. (2019). Evaluation of the virucidal efficacy of disinfectant wipes with a test method simulating practical conditions. Antimicrob. Resist. Infect. Control.

[32] Eterpi M., McDonnell G., Thomas V. (2010). Virucidal Activity of Disinfectants against Parvoviruses and Reference Viruses. Appl. Biosaf..

[33] Morin T., Martin H., Soumet C., Fresnel R., Lamaudière S., Le Sauvage A., Deleurme K., Maris P. (2015). Comparison of the virucidal efficacy of peracetic acid, potassium monopersulphate and sodium hypochlorite on bacteriophages P001 and MS2. J. Appl. Microbiol..

[34] Maillard J.-Y., Hann A., Baubet V., Perrin R. (1998). Efficacy and mechanisms of action of sodium hypochlorite on Pseudomonas aeruginosa PAO1 phage F116. J. Appl. Microbiol..

[35] OIE Report of the Meeting of the OIE Aquatic Animal Health Standards Commission. Presented at the OIE Headquarters.

[36] United States Department of Agriculture (2020). Disinfectants approved for use against African swine fever virus in farm settings. https://www.aphis.usda.gov/animal_health/emergency_management/downloads/asf-virus-disinfectants.pdf.

[37] Plowright W., Parker J. (1967). The stability of African swine fever virus with particular reference to heat and pH inactivation. Arch. Virol..

[38] Maris P. (1995). Modes of action of disinfectants. Rev. Sci. et Tech. de l’OIE.

